# Crystal structure of *trans*-di­aqua­bis­(4-cyano­benzoato-κ*O*)bis­(nicotinamide-κ*N* ^1^)cobalt(II)

**DOI:** 10.1107/S2056989015008270

**Published:** 2015-04-30

**Authors:** Gülçin Şefiye Aşkın, Hacali Necefoğlu, Gamze Yılmaz Nayir, Raziye Çatak Çelik, Tuncer Hökelek

**Affiliations:** a Department of Physics, Hacettepe University, 06800 Beytepe, Ankara, Turkey; b Department of Chemistry, Kafkas University, 36100 Kars, Turkey; c International Scientific Research Centre, Baku State University, 1148 Baku, Azerbaijan; d Scientific and Technological Application and Research Center, Aksaray University, 68100 Aksaray, Turkey

**Keywords:** crystal structure, cobalt(II), transition metal complexes of benzoic acid and nicotinamide derivatives

## Abstract

In the title complex, [Co(C_8_H_4_NO_2_)_2_(C_6_H_6_N_2_O)_2_(H_2_O)_2_], the Co^II^ atom is located on an inversion centre and is coordinated by two 4-cyano­benzoate (CNB) anions, two nicotinamide (NA) ligands and two water mol­ecules. The four O atoms in the equatorial plane form a slightly distorted square-planar arrangement, while the slightly distorted octa­hedral coordination sphere is completed by the two N atoms of the NA ligands in the axial positions. The dihedral angle between the carboxyl­ate group and the adjacent benzene ring is 22.11 (15)°, while the pyridine and benzene rings are oriented at a dihedral angle of 89.98 (5)°. In the crystal, inter­molecular N—H⋯O and O—H⋯O hydrogen bonds link the mol­ecules, enclosing *R*_2_^2^(8) and *R*_4_^4^(8) ring motifs, forming layers parallel to (100). The layers are linked *via* C—H⋯O and C—H⋯N hydrogen bonds, resulting in a three-dimensional network. A weak C—H⋯π inter­action is also observed.

## Abstract 2

In the title complex, the Co^II^ atom is located on an inversion centre and is coordinated by two 4-cyano­benzoate (CNB) anions, two nicotinamide (NA) ligands and two water mol­ecules. The four O atoms in the equatorial plane form a slightly distorted square-planar arrangement, while the slightly distorted octa­hedral coordination sphere is completed by the two N atoms of the NA ligands in the axial positions.

## Chemical context   

Nicotinamide (NA) is one form of niacin. A deficiency of this vitamin leads to loss of copper from the body, known as pellagra disease. Victims of pellagra show unusually high serum and urinary copper levels (Krishnamachari, 1974 ▸). The nicotinic acid derivative *N*,*N*-di­ethyl­nicotinamide (DENA) is an important respiratory stimulant (Bigoli *et al.*, 1972 ▸). Trans­ition metal complexes with biochemical-relevant mol­ecules show inter­esting physical and/or chemical properties, through which they may find applications in biological systems (Antolini *et al.*, 1982 ▸). Some benzoic acid derivatives, such as 4-amino­benzoic acid, have been extensively reported in coordination chemistry, as bifunctional organic ligands, due to the varieties of their coordination modes (Chen & Chen, 2002 ▸; Amiraslanov *et al.*, 1979 ▸; Hauptmann *et al.*, 2000 ▸).
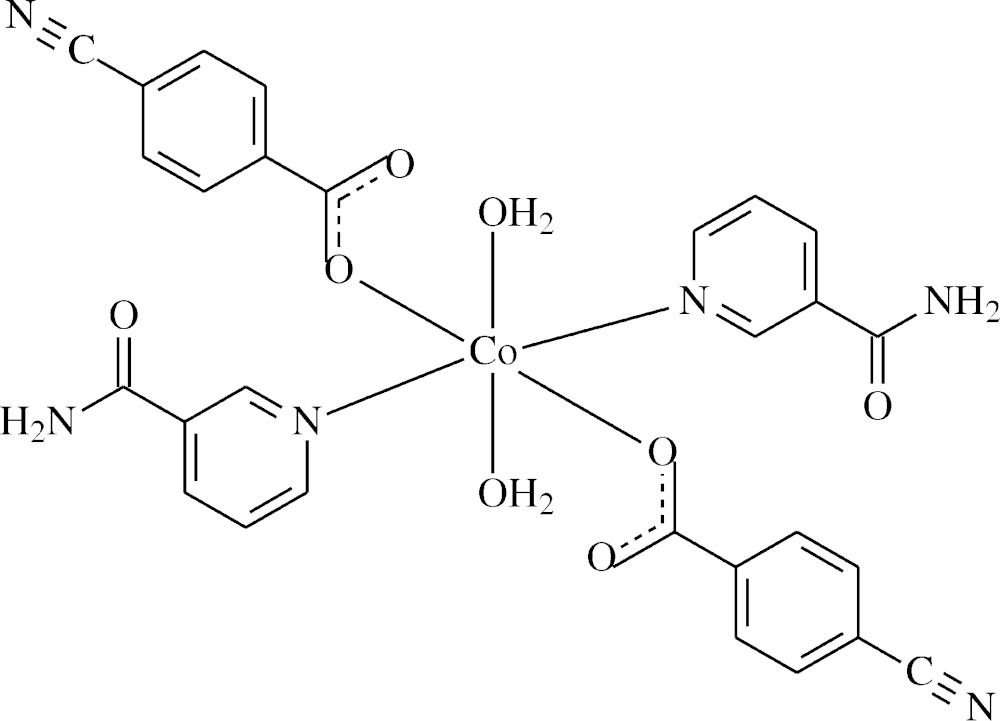


The structure–function–coordination relationships of the aryl­carboxyl­ate ion in Zn^II^ complexes of benzoic acid derivatives change depending on the nature and position of the substituted groups on the benzene ring, the nature of the additional ligand mol­ecule or solvent, and the pH and temperature of synthesis (Shnulin *et al.*, 1981 ▸; Nadzhafov *et al.*, 1981 ▸; Antsyshkina *et al.*, 1980 ▸; Adiwidjaja *et al.*, 1978 ▸). When pyridine and its derivatives are used instead of water mol­ecules, the structure is completely different (Catterick *et al.*, 1974 ▸). In this context, we synthesized the Co^II^-containing title compound, *trans*-di­aqua­bis­(4-cyano­benzoato-κ*O*)bis­(nico­tinamide-κ*N* ^1^)cobalt(II), [Co(C_8_H_4_O_2_N)_2_(C_6_H_6_N_2_O)_2_(H_2_O)_2_], and report herein its crystal structure.

## Structural commentary   

**Figure 1:**
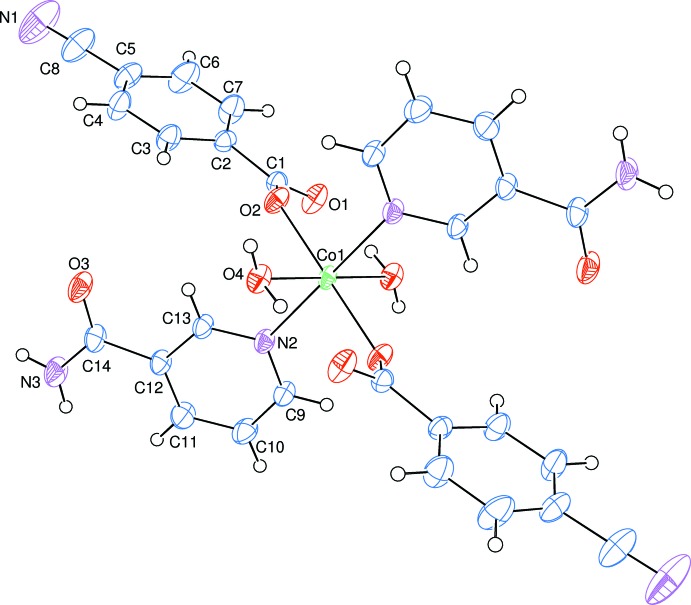
The mol­ecular structure of the title compound with the atom-numbering scheme. Displacement ellipsoids are drawn at the 50% probability level. Symmetry-related atoms are defined by symmetry code −*x* + 1, −*y* + 1, −*z* + 1.

In the mononuclear title complex, the Co^II^ atom is located on an inversion centre and is coordinated by two 4-cyano­benzoate (CNB) anions, two nicotinamide (NA) ligands and two water mol­ecules, with all ligands coordinating in a monodentate manner (Fig. 1 ▸).

The two symmetry-related carboxyl­ate O atoms (O2 and O2^i^) and the two symmetry-related water O atoms (O4 and O4^i^) form a slightly distorted square-planar arrangement, while the slightly distorted octa­hedral coordination sphere is completed by the two symmetry-related N atoms (N2 and N2^i^) of the two NA ligands in the axial positions [symmetry code: (i) −*x* + 1, −*y* + 1, −*z* + 1] (Fig. 1 ▸).

The very similar C1—O1 [1.254 (2) Å] and C1—O2 [1.256 (2) Å], bond lengths of the carboxyl­ate group indicate delocalized bonding arrangements, rather than localized single and double bonds. The Co—O bond lengths are 2.0835 (12) Å (for benzoate oxygen atoms) and 2.1350 (13) Å (for water oxygen atoms), and the Co—N bond length is 2.1390 (15) Å, close to standard values. The Co1 atom lies 0.3921 (1) Å above the planar (O1/O2/C1) carboxyl­ate group. The O—Co—O and O—Co—N bond angles deviate only slightly from ideal values, with average values of 90 (3)° and 90 (2)°, respectively.

The dihedral angle between the planar carboxyl­ate group (O1/O2/C1) and the adjacent benzene ring [*A* (C2–C7)] is 22.11 (15)°, while the benzene and pyridine [*B* (N2/C9–C13)] rings are oriented at a dihedral angle of 89.98 (5)°.

## Supra­molecular features   

**Figure 2:**
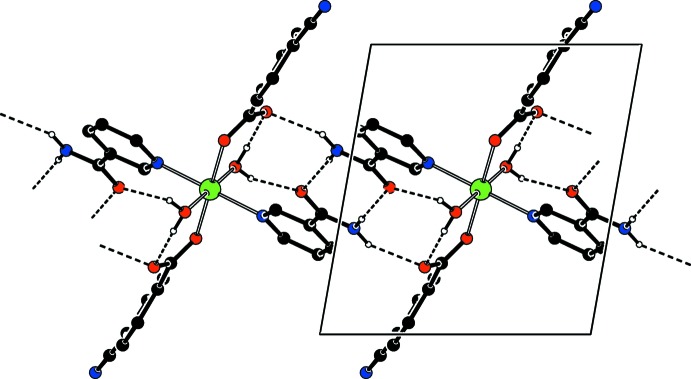
Part of the crystal structure viewed down [100], where the *b* axis is horizontal and the *c* axis is vertical. Inter­molecular N—H⋯O and O—H⋯O hydrogen bonds are shown as dashed lines. Non-bonding H atoms have been omitted for clarity.

In the crystal, N—H⋯O_c_ (c = carboxyl­ate), N—H⋯O_n_ (n = nicotinamide), O—H_w_⋯O_c_ (w = water) and O—H_w_⋯O_n_ hydrogen bonds (Table 1 ▸) link the mol­ecules, enclosing 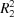
(8) and 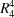
(8) ring motifs (Bernstein *et al.*, 1995 ▸), forming layers parallel to (100) (Fig. 2 ▸). The layers are linked *via* C—H_cnb_⋯O_c_ (cnb = cyano­benzoate) and C—H_n_⋯N_cnb_ hydrogen bonds (Table 1 ▸), resulting in a three-dimensional network. A weak C—H⋯π inter­action is also observed.

## Synthesis and crystallization   

The title compound was prepared by the reaction of CoSO_4_·7H_2_O (1.41 g, 5 mmol) in H_2_O (50 ml) and nicotinamide (1.22 g, 50 mmol) in H_2_O (50 ml) with sodium 4-cyano­benzoate (1.69 g, 10 mmol) in H_2_O (100 ml). The mixture was filtered and set aside to crystallize at ambient temperature for several days, giving pink-coloured single crystals.

## Refinement   

The experimental details including the crystal data, data collection and refinement are summarized in Table 2 ▸. Atoms H31 and H32 (for NH_2_) and H41 and H42 (for H_2_O) were located in a difference Fourier map and were refined freely. The aromatic C-bound H atoms were positioned geometrically with C—H = 0.93 Å, and constrained to ride on their parent atoms, with *U*_iso_(H) = 1.2*U*_eq_(C). The highest electron density and the deepest hole were found 0.80 Å and 0.83 Å, respectively, from Co1.

## Supplementary Material

Crystal structure: contains datablock(s) I, global. DOI: 10.1107/S2056989015008270/wm5151sup1.cif

Structure factors: contains datablock(s) I. DOI: 10.1107/S2056989015008270/wm5151Isup2.hkl

CCDC reference: 1061935

Additional supporting information:  crystallographic information; 3D view; checkCIF report

## Figures and Tables

**Table 1:** Hydrogen-bond geometry (, ) *Cg*2 is the centroid of the C9C13,N2 ring.

*D*H*A*	*D*H	H*A*	*D* *A*	*D*H*A*
N3H31O3^i^	0.84(3)	2.09(3)	2.914(3)	166(3)
N3H32O1^ii^	0.87(3)	2.13(3)	2.910(3)	148(3)
O4H41O1^iii^	0.85(3)	1.82(3)	2.658(2)	166(3)
O4H42O3^iv^	0.80(3)	2.11(3)	2.877(2)	161(2)
C4H4O1^v^	0.93	2.38	3.302(3)	173
C9H9N1^vi^	0.93	2.54	3.305(5)	140
C6H6*Cg*2^vii^	0.93	2.76	3.691(2)	176

Symmetry codes: (i) 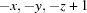; (ii) 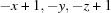; (iii) 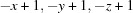; (iv) 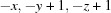; (v) 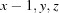; (vi) 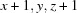; (vii) 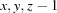.

**Table 2:** Experimental details

Crystal data	
Chemical formula	[Co(C_8_H_4_NO_2_)_2_(C_6_H_6_N_2_O)_2_(H_2_O)_2_]
*M*_r_	631.46
Crystal system, space group	Triclinic, *P* 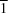
Temperature (K)	296
*a*, *b*, *c* ()	7.6474(3), 9.9266(4), 10.2782(4)
, , ()	78.680(2), 84.200(3), 71.556(2)
*V* (^3^)	725.13(5)
*Z*	1
Radiation type	Mo *K*
(mm^1^)	0.65
Crystal size (mm)	0.43 0.29 0.16

Data collection	
Diffractometer	Bruker SMART BREEZE CCD
Absorption correction	Multi-scan (*SADABS*; Bruker, 2012 ▸)
*T*_min_, *T*_max_	0.797, 0.901
No. of measured, independent and observed [*I* > 2(*I*)] reflections	16533, 3639, 3419
*R*_int_	0.036
(sin /)_max_ (^1^)	0.672

Refinement	
*R*[*F* ^2^ > 2(*F* ^2^)], *wR*(*F* ^2^), *S*	0.044, 0.119, 1.07
No. of reflections	3639
No. of parameters	212
H-atom treatment	H atoms treated by a mixture of independent and constrained refinement
_max_, _min_ (e ^3^)	0.94, 0.49

Computer programs: *APEX2* and *SAINT* (Bruker, 2012 ▸), *SHELXS97* and *SHELXL97* (Sheldrick, 2008 ▸), *ORTEP-3 for Windows* (Farrugia, 2012 ▸) and *WinGX* (Farrugia, 2012 ▸) and *PLATON* (Spek, 2009 ▸).

## supplementary crystallographic information

### Crystal data

**Table:**

[Co(C_8_H_4_NO_2_)_2_(C_6_H_6_N_2_O)_2_(H_2_O)_2_]	*Z* = 1
*M**_r_* = 631.46	*F*(000) = 325
Triclinic, *P*1	*D*_x_ = 1.446 Mg m^−^^3^
Hall symbol: -P 1	Mo *K*α radiation, λ = 0.71073 Å
*a* = 7.6474 (3) Å	Cell parameters from 9898 reflections
*b* = 9.9266 (4) Å	θ = 2.2–28.6°
*c* = 10.2782 (4) Å	µ = 0.65 mm^−^^1^
α = 78.680 (2)°	*T* = 296 K
β = 84.200 (3)°	Prism, translucent light pink
γ = 71.556 (2)°	0.43 × 0.29 × 0.16 mm
*V* = 725.13 (5) Å^3^	

### Data collection

**Table:**

Bruker SMART BREEZE CCD diffractometer	3639 independent reflections
Radiation source: fine-focus sealed tube	3419 reflections with *I* > 2σ(*I*)
Graphite monochromator	*R*_int_ = 0.036
φ and ω scans	θ_max_ = 28.6°, θ_min_ = 2.0°
Absorption correction: multi-scan (*SADABS*; Bruker, 2012)	*h* = −10→10
*T*_min_ = 0.797, *T*_max_ = 0.901	*k* = −12→13
16533 measured reflections	*l* = −13→13

### Refinement

**Table:**

Refinement on *F*^2^	Primary atom site location: structure-invariant direct methods
Least-squares matrix: full	Secondary atom site location: difference Fourier map
*R*[*F*^2^ > 2σ(*F*^2^)] = 0.044	Hydrogen site location: inferred from neighbouring sites
*wR*(*F*^2^) = 0.119	H atoms treated by a mixture of independent and constrained refinement
*S* = 1.07	*w* = 1/[σ^2^(*F*_o_^2^) + (0.0721*P*)^2^ + 0.2871*P*] where *P* = (*F*_o_^2^ + 2*F*_c_^2^)/3
3639 reflections	(Δ/σ)_max_ < 0.001
212 parameters	Δρ_max_ = 0.94 e Å^−^^3^
0 restraints	Δρ_min_ = −0.49 e Å^−^^3^

### Special details

**Table:**

Geometry. All esds (except the esd in the dihedral angle between two l.s. planes) are estimated using the full covariance matrix. The cell esds are taken into account individually in the estimation of esds in distances, angles and torsion angles; correlations between esds in cell parameters are only used when they are defined by crystal symmetry. An approximate (isotropic) treatment of cell esds is used for estimating esds involving l.s. planes.
Refinement. Refinement of F^2^ against ALL reflections. The weighted R-factor wR and goodness of fit S are based on F^2^, conventional R-factors R are based on F, with F set to zero for negative F^2^. The threshold expression of F^2^ > 2sigma(F^2^) is used only for calculating R-factors(gt) etc. and is not relevant to the choice of reflections for refinement. R-factors based on F^2^ are statistically about twice as large as those based on F, and R- factors based on ALL data will be even larger.

### Fractional atomic coordinates and isotropic or equivalent isotropic displacement parameters (Å^2^)

**Table:**

	*x*	*y*	*z*	*U*_iso_*/*U*_eq_	
Co1	0.5000	0.5000	0.5000	0.02573 (12)	
O1	0.6366 (2)	0.34341 (18)	0.23257 (16)	0.0459 (4)	
O2	0.38784 (18)	0.47948 (14)	0.33110 (13)	0.0347 (3)	
O3	0.0668 (2)	0.16139 (15)	0.50697 (18)	0.0476 (4)	
O4	0.22430 (19)	0.57799 (15)	0.57811 (16)	0.0362 (3)	
H41	0.250 (4)	0.608 (3)	0.644 (3)	0.064 (9)*	
H42	0.147 (4)	0.643 (3)	0.538 (2)	0.038 (6)*	
N1	−0.0584 (5)	0.1958 (4)	−0.1318 (4)	0.1083 (13)	
N2	0.4952 (2)	0.28815 (16)	0.59167 (15)	0.0303 (3)	
N3	0.1614 (3)	−0.0568 (2)	0.6345 (2)	0.0473 (5)	
H31	0.080 (4)	−0.079 (3)	0.602 (3)	0.063 (9)*	
H32	0.238 (4)	−0.121 (3)	0.690 (3)	0.054 (8)*	
C1	0.4662 (2)	0.40272 (19)	0.24567 (17)	0.0300 (3)	
C2	0.3451 (2)	0.37166 (19)	0.15590 (17)	0.0298 (3)	
C3	0.1622 (3)	0.3852 (2)	0.19215 (19)	0.0382 (4)	
H3	0.1098	0.4240	0.2675	0.046*	
C4	0.0562 (3)	0.3414 (3)	0.1171 (2)	0.0457 (5)	
H4	−0.0661	0.3486	0.1430	0.055*	
C5	0.1336 (3)	0.2869 (3)	0.0037 (2)	0.0458 (5)	
C6	0.3143 (4)	0.2788 (3)	−0.0368 (2)	0.0570 (7)	
H6	0.3641	0.2460	−0.1152	0.068*	
C7	0.4203 (3)	0.3198 (3)	0.0398 (2)	0.0470 (5)	
H7	0.5424	0.3126	0.0137	0.056*	
C8	0.0255 (4)	0.2372 (4)	−0.0727 (3)	0.0680 (8)	
C9	0.6293 (2)	0.1956 (2)	0.66831 (19)	0.0339 (4)	
H9	0.7280	0.2252	0.6841	0.041*	
C10	0.6261 (3)	0.0585 (2)	0.7244 (2)	0.0428 (5)	
H10	0.7210	−0.0033	0.7772	0.051*	
C11	0.4798 (3)	0.0138 (2)	0.7013 (2)	0.0419 (4)	
H11	0.4756	−0.0786	0.7379	0.050*	
C12	0.3394 (2)	0.10851 (18)	0.62286 (18)	0.0307 (4)	
C13	0.3539 (2)	0.24444 (18)	0.57084 (18)	0.0304 (3)	
H13	0.2601	0.3088	0.5185	0.037*	
C14	0.1776 (3)	0.07268 (19)	0.5848 (2)	0.0352 (4)	

### Atomic displacement parameters (Å^2^)

**Table:**

	*U*^11^	*U*^22^	*U*^33^	*U*^12^	*U*^13^	*U*^23^
Co1	0.02515 (17)	0.02306 (18)	0.03406 (19)	−0.01019 (12)	−0.00572 (12)	−0.00978 (12)
O1	0.0315 (7)	0.0592 (9)	0.0516 (8)	−0.0103 (6)	−0.0066 (6)	−0.0234 (7)
O2	0.0355 (6)	0.0333 (6)	0.0400 (7)	−0.0084 (5)	−0.0111 (5)	−0.0157 (5)
O3	0.0445 (8)	0.0303 (7)	0.0743 (11)	−0.0156 (6)	−0.0241 (7)	−0.0061 (7)
O4	0.0290 (6)	0.0348 (7)	0.0464 (8)	−0.0075 (5)	−0.0060 (6)	−0.0120 (6)
N1	0.104 (2)	0.126 (3)	0.121 (3)	−0.030 (2)	−0.052 (2)	−0.065 (2)
N2	0.0304 (7)	0.0275 (7)	0.0370 (7)	−0.0115 (6)	−0.0065 (6)	−0.0082 (6)
N3	0.0515 (11)	0.0305 (8)	0.0691 (13)	−0.0229 (8)	−0.0187 (9)	−0.0046 (8)
C1	0.0332 (8)	0.0290 (8)	0.0312 (8)	−0.0130 (7)	−0.0068 (6)	−0.0040 (6)
C2	0.0342 (8)	0.0291 (8)	0.0288 (8)	−0.0102 (7)	−0.0060 (6)	−0.0078 (6)
C3	0.0361 (9)	0.0491 (11)	0.0349 (9)	−0.0137 (8)	−0.0023 (7)	−0.0184 (8)
C4	0.0368 (10)	0.0620 (14)	0.0475 (11)	−0.0207 (9)	−0.0054 (8)	−0.0196 (10)
C5	0.0500 (11)	0.0505 (12)	0.0437 (11)	−0.0140 (9)	−0.0159 (9)	−0.0190 (9)
C7	0.0396 (10)	0.0679 (15)	0.0403 (10)	−0.0181 (10)	0.0037 (8)	−0.0253 (10)
C6	0.0523 (13)	0.0836 (18)	0.0428 (11)	−0.0148 (12)	−0.0034 (9)	−0.0377 (12)
C8	0.0649 (16)	0.0794 (19)	0.0706 (17)	−0.0168 (14)	−0.0256 (13)	−0.0357 (15)
C9	0.0295 (8)	0.0345 (9)	0.0404 (9)	−0.0108 (7)	−0.0076 (7)	−0.0083 (7)
C10	0.0382 (10)	0.0349 (10)	0.0529 (12)	−0.0071 (8)	−0.0171 (8)	−0.0012 (8)
C11	0.0456 (11)	0.0248 (8)	0.0561 (12)	−0.0123 (8)	−0.0133 (9)	−0.0006 (8)
C12	0.0331 (8)	0.0241 (8)	0.0395 (9)	−0.0111 (6)	−0.0046 (7)	−0.0106 (7)
C13	0.0311 (8)	0.0240 (7)	0.0399 (9)	−0.0104 (6)	−0.0091 (7)	−0.0069 (7)
C14	0.0361 (9)	0.0270 (8)	0.0490 (10)	−0.0139 (7)	−0.0043 (7)	−0.0133 (7)

### Geometric parameters (Å, º)

**Table:**

Co1—O2	2.0835 (12)	C2—C7	1.391 (3)
Co1—O2^i^	2.0835 (12)	C3—C4	1.387 (3)
Co1—O4	2.1350 (13)	C3—H3	0.9300
Co1—O4^i^	2.1350 (13)	C4—C5	1.381 (3)
Co1—N2	2.1390 (15)	C4—H4	0.9300
Co1—N2^i^	2.1390 (15)	C5—C6	1.384 (4)
O1—C1	1.254 (2)	C5—C8	1.444 (3)
O2—C1	1.256 (2)	C6—H6	0.9300
O3—C14	1.234 (2)	C7—C6	1.380 (3)
O4—H41	0.85 (3)	C7—H7	0.9300
O4—H42	0.80 (3)	C9—C10	1.378 (3)
N1—C8	1.136 (4)	C9—H9	0.9300
N2—C9	1.341 (2)	C10—H10	0.9300
N2—C13	1.335 (2)	C11—C10	1.383 (3)
N3—C14	1.326 (3)	C11—H11	0.9300
N3—H31	0.84 (3)	C12—C11	1.388 (3)
N3—H32	0.87 (3)	C12—C13	1.385 (2)
C1—C2	1.506 (2)	C12—C14	1.497 (2)
C2—C3	1.381 (3)	C13—H13	0.9300
			
O2—Co1—O2^i^	180.0	C4—C3—H3	119.7
O2—Co1—O4	87.59 (6)	C3—C4—H4	120.3
O2^i^—Co1—O4	92.41 (6)	C5—C4—C3	119.5 (2)
O2—Co1—O4^i^	92.41 (6)	C5—C4—H4	120.3
O2^i^—Co1—O4^i^	87.59 (6)	C4—C5—C6	120.46 (19)
O4—Co1—O4^i^	180.000 (1)	C4—C5—C8	119.7 (2)
O2—Co1—N2	89.99 (6)	C6—C5—C8	119.8 (2)
O2^i^—Co1—N2	90.01 (5)	C5—C6—H6	120.1
O2—Co1—N2^i^	90.01 (5)	C7—C6—C5	119.8 (2)
O2^i^—Co1—N2^i^	89.99 (6)	C7—C6—H6	120.1
O4—Co1—N2	87.40 (6)	C2—C7—H7	119.9
O4^i^—Co1—N2	92.60 (6)	C6—C7—C2	120.3 (2)
O4—Co1—N2^i^	92.60 (6)	C6—C7—H7	119.9
O4^i^—Co1—N2^i^	87.40 (6)	N1—C8—C5	178.9 (4)
N2^i^—Co1—N2	180.00 (4)	N2—C9—C10	122.33 (17)
C1—O2—Co1	127.68 (11)	N2—C9—H9	118.8
Co1—O4—H41	97 (2)	C10—C9—H9	118.8
Co1—O4—H42	122.7 (18)	C9—C10—C11	119.16 (17)
H42—O4—H41	107 (3)	C9—C10—H10	120.4
C9—N2—Co1	123.03 (12)	C11—C10—H10	120.4
C13—N2—Co1	118.91 (12)	C10—C11—C12	119.24 (18)
C13—N2—C9	118.05 (16)	C10—C11—H11	120.4
C14—N3—H31	116 (2)	C12—C11—H11	120.4
C14—N3—H32	123.3 (19)	C11—C12—C14	124.94 (17)
H32—N3—H31	120 (3)	C13—C12—C11	117.65 (16)
O1—C1—O2	125.70 (17)	C13—C12—C14	117.35 (16)
O1—C1—C2	116.72 (16)	N2—C13—C12	123.58 (16)
O2—C1—C2	117.48 (15)	N2—C13—H13	118.2
C3—C2—C1	120.48 (16)	C12—C13—H13	118.2
C3—C2—C7	119.43 (17)	O3—C14—N3	122.08 (18)
C7—C2—C1	119.95 (17)	O3—C14—C12	119.90 (17)
C2—C3—C4	120.52 (18)	N3—C14—C12	118.00 (18)
C2—C3—H3	119.7		
			
O4—Co1—O2—C1	−159.74 (16)	O2—C1—C2—C7	163.80 (19)
O4^i^—Co1—O2—C1	20.26 (16)	C1—C2—C3—C4	−172.77 (19)
N2—Co1—O2—C1	−72.35 (16)	C7—C2—C3—C4	2.9 (3)
N2^i^—Co1—O2—C1	107.65 (16)	C1—C2—C7—C6	174.2 (2)
O2—Co1—N2—C9	141.91 (15)	C3—C2—C7—C6	−1.6 (4)
O2^i^—Co1—N2—C9	−38.09 (15)	C2—C3—C4—C5	−1.5 (4)
O2—Co1—N2—C13	−37.22 (14)	C3—C4—C5—C6	−1.4 (4)
O2^i^—Co1—N2—C13	142.78 (14)	C3—C4—C5—C8	178.2 (2)
O4—Co1—N2—C9	−130.50 (15)	C4—C5—C6—C7	2.8 (4)
O4^i^—Co1—N2—C9	49.50 (15)	C8—C5—C6—C7	−176.8 (3)
O4—Co1—N2—C13	50.36 (14)	C2—C7—C6—C5	−1.3 (4)
O4^i^—Co1—N2—C13	−129.64 (14)	N2—C9—C10—C11	0.0 (3)
Co1—O2—C1—O1	−13.7 (3)	C12—C11—C10—C9	−0.4 (3)
Co1—O2—C1—C2	162.49 (12)	C13—C12—C11—C10	0.2 (3)
Co1—N2—C9—C10	−178.63 (15)	C14—C12—C11—C10	177.2 (2)
C13—N2—C9—C10	0.5 (3)	C11—C12—C13—N2	0.3 (3)
Co1—N2—C13—C12	178.47 (14)	C14—C12—C13—N2	−176.86 (17)
C9—N2—C13—C12	−0.7 (3)	C11—C12—C14—O3	−174.9 (2)
O1—C1—C2—C3	156.08 (19)	C11—C12—C14—N3	3.4 (3)
O1—C1—C2—C7	−19.6 (3)	C13—C12—C14—O3	2.1 (3)
O2—C1—C2—C3	−20.5 (3)	C13—C12—C14—N3	−179.63 (19)

Symmetry code: (i) −*x*+1, −*y*+1, −*z*+1.

### Hydrogen-bond geometry (Å, º)

Cg2 is the centroid of the C9–C13,N2 ring.

**Table:**

*D*—H···*A*	*D*—H	H···*A*	*D*···*A*	*D*—H···*A*
N3—H31···O3^ii^	0.84 (3)	2.09 (3)	2.914 (3)	166 (3)
N3—H32···O1^iii^	0.87 (3)	2.13 (3)	2.910 (3)	148 (3)
O4—H41···O1^i^	0.85 (3)	1.82 (3)	2.658 (2)	166 (3)
O4—H42···O3^iv^	0.80 (3)	2.11 (3)	2.877 (2)	161 (2)
C4—H4···O1^v^	0.93	2.38	3.302 (3)	173
C9—H9···N1^vi^	0.93	2.54	3.305 (5)	140
C6—H6···*Cg*2^vii^	0.93	2.76	3.691 (2)	176

Symmetry codes: (i) −*x*+1, −*y*+1, −*z*+1; (ii) −*x*, −*y*, −*z*+1; (iii) −*x*+1, −*y*, −*z*+1; (iv) −*x*, −*y*+1, −*z*+1; (v) *x*−1, *y*, *z*; (vi) *x*+1, *y*, *z*+1; (vii) *x*, *y*, *z*−1.
